# Similarities in Bacterial Uropathogens and Their Antimicrobial Susceptibility Profile in Diabetics and Their Non-Diabetic Caregivers at a National Diabetes Management and Research Centre, Accra-Ghana

**DOI:** 10.3390/diseases10040124

**Published:** 2022-12-12

**Authors:** Enid Owusu, Henrietta Adjei, Emmanuel Afutu

**Affiliations:** 1 Department of Medical Laboratory Sciences, School of Biomedical and Allied Health Sciences, University of Ghana, Accra 00233, Ghana; 2 Department of Medical Microbiology, School of Biomedical and Allied Health Sciences, University of Ghana, Accra 00233, Ghana

**Keywords:** antibiotic, bacteria, diabetes, resistance, susceptibility, urinary tract, UTI

## Abstract

Background: Diabetes mellitus has some damaging effects on the genitourinary system and has been found to have adverse effects on the host immune system, resulting in higher risk of infections including urinary tract infections (UTI). Incidences of UTI caused by bacteria have been increasingly reported globally and the abuse of antibiotics leading to evolving resistant strains of antibiotics is a public health challenge for the management of this condition. Information on the difference in types of bacterial agents causing UTI in diabetic patients and non-diabetic individuals, and their susceptibility profiles, will facilitate effective management among these groups of patients. Therefore, this study aimed at determining the difference in prevalence of UTI, the causative bacteria, and their antimicrobial susceptibility profiles in diabetic patients and non-diabetic individuals at a diabetes management center. Methods: This was a prospective cross-sectional study conducted amongst 100 diagnosed diabetic patients and 100 non-diabetic individuals. Urine sample was collected aseptically and analyzed microbiologically for the presence of urinary tract bacterial pathogens. Drug susceptibility testing was conducted on the isolates by the Kirby Bauer method to ascertain the antibiotic susceptibility patterns. Results: Among the diabetic and non-diabetic individuals, urinary tract bacterial pathogens were observed in 28.0% and 26.0% of samples, respectively. The organisms were in the following proportions for diabetic and non-diabetic individuals, respectively: *E. coli* (14/28, 50% and 8/26, 30.7%), *S. aureus* (2/28, 7.1% and 4/26, 15.4%), *K. pneumoniae* (4/28, 14.3% and 8/26, 30.7%), *K. ozoenae* (2/28, 7.14% and 0/26, 0%), *K. oxytoca* (0/28, 0% and 4/26, 15.4%) and *C. urealyticum* (6/28, 21.4% and 2/26, 7.69%). The difference between the proportions of bacteria isolated was, however, not statistically significant (*p*-value = 0.894). Bacteria isolated from both diabetic and non-diabetic individuals were highly susceptible to most of the antibiotics tested, especially nitrofurantoin, cefuroxime, ceftriaxone, and cefotaxime. Conclusion: This study has shown that similarities exist in prevalence of UTI, the causative bacteria, and their antimicrobial susceptibility patterns amongst diabetic patients and non-diabetic individuals at a diabetes management center. These data will help in the management of UTI among these individuals.

## 1. Introduction

Diabetes mellitus (DM) is a metabolic disease characterized by high levels of blood glucose due to the effect of impaired insulin utilization, abnormal insulin secretion, or both [1]. Symptoms of high blood glucose include frequent urination, increased thirst, and increased hunger [1]. DM is responsible for significant ill health, especially if it is not recognized early or when not treated [2]. Although it causes acute complications such as diabetic ketoacidosis and nonketotic hyperosmolar coma, it has also been considered as an influencing factor for urinary tract infections [3]. Both type 1 and type 2 diabetes have been linked with complications affecting various organs in the body. This may be due to damage in small vessels resulting in nephropathy, retinopathy, and neuropathy, and macroangiopathy, i.e., an accelerated arteriosclerosis, leading to cardiovascular morbidity and death [4,5]. Complications due to chronic diabetes affect quality of life and/or life expectancy and increase the burden on both the individual patient as well as society at large, through high costs of health care and loss of productivity [6].

Diabetes is a major cause of adult disability and death in Ghana [7]. The prevalence of diabetes has increased from 0.2% in the late 1950s (among a community of men in Ho) to 6.0% in 2009 (among a community of men in Kumasi) [7]. In Accra (where the current study was conducted), a prevalence of 6.4% was reported in a mixed community in the late 1990s and 9.1% among civil servants in 2006 [7]. Overall, Asamoah-Boaheng and colleagues [8] have described the prevalence of diabetes mellitus among adult Ghanaians to be at 6.46% based on an inverse-variance random-effects model.

Urinary tract infection (UTI) is one of the commonest infections associated with diabetic patients globally, especially in developing countries [9]. DM has a greater effect on the genitourinary tract system and increases the risk of UTI as the immune system becomes compromised and opportunistic bacteria take advantage of this condition to cause UTIs [10]. Some of these bacteria include *Escherichia coli*, *Klebsiella pneumoniae* and *Proteus* mirabilis, which possess virulent factors that enable them to colonize urinary epithelial cells [11]. In Ghana (specifically Accra where this study was conducted), the most common uropathogen associated with community-acquired UTI is *Escherichia coli*, followed by *Klebseilla* sp. [12]. Prevalence of resistance among patients is highest for piperacillin and amoxicillin/clavulanic acid and lowest for amikacin. Pregnant women constitute the key risk population of UTI in Accra, and amikacin remains a suitable drug for the treatment of febrile UTI [12].

The primary etiological agents of UTIs in diabetics are Gram-negative bacteria; however, Gram-positive bacteria may also be involved, with the common uropathogens being *Escherichia coli*, *Staphylococcus saprophyticus*, *Klebsiella pneumoniae*, *Proteus mirabilis*, *Enterococcus* species, *Pseudomonas aeruginosa* and group B *streptococcus*. In a study by Worku et al., [13] five species of bacteria were isolated in the urine of diabetics with *E. coli* being the leading uropathogen, followed by *K. pneumoniae*. That study showed a high prevalence of drug resistance to common antimicrobials, particularly to co-trimoxazole, ciprofloxacin, doxycycline, ampicillin, amoxicillin-clavulanate, cefuroxime and penicillin [13]. 

In another study, Akbar [14] reported on urinary tract infection in diabetics and non-diabetic individuals and observed that *Escherichia coli* and *Pseudomonas* species were both found in diabetics and non-diabetics, with these organisms (*Escherichia coli* and pseudomonas in both groups) showing resistance to ampicillin and sensitivity to aminoglycoside and ciprofloxacin.

Furthermore, Ramrakhia et al. [15] have showed that the overall incidence of UTI in participants from a diabetic group was significantly higher than those in the non-diabetic group, with *Escherichia coli* being the most common organism in both the diabetic and non-diabetic groups. They also observed that the frequency of Klebsiella was considerably higher in the participants with diabetes, but it was not significant [15].

Higher incidence of UTI in diabetic patients has been attributed to reasons such as damage to nerves caused by high blood glucose levels, which in turn affects the ability of the bladder to sense the presence of urine, as well as high glucose levels in urine, encouraging the growth of the bacteria [16,17,18]. Furthermore, the chemical composition of urine is said to become altered, leading to it supporting the growth of these opportunistic bacteria [19]. 

In diabetic patients, UTIs can cause numerous complications, ranging from dysuria to organ damage, as well as complicated UTI (pyelonephritis) [20]. Bacterial pathogens that are the most common causes of UTIs in patients with diabetes include *Escherichia coli*, *Klebsiella* spp., *Proteus mirabilis*, *Pseudomonas* spp. and *Staphylococcus aureus* [20,21]. Over the years, certain bacterial pathogens have developed varying degrees of resistance to some of the potent antibiotics, and this as a result has led to difficulty in the management of UTIs [22], especially in older individuals [23]. There is also evidence that patients with diabetes have an increased asymptomatic bacteriuria, making the management of UTIs more challenging than those without [24]. Urinary tract infections make it difficult to control blood sugar in diabetic patients, which increases the need for blood sugar monitoring, reduces the quality of life, and imposes significant treatment costs on the patient [25]. However, according to Salari [26], there is a difference of opinion regarding the effect of diabetes duration and blood sugar control on UTI. 

Meanwhile, studies continue to investigate incidence of UTI among DM patients [27,28]. For example, a cross-sectional study by Hamdan et al. [27] presented information on urinary tract infections and antimicrobial sensitivity among diabetic patients. In Ethiopia, bacteriuria among symptomatic diabetic patients has been reported [29]. UTI prevalence rates of 22% and 38.3% have been reported in diabetes patients in South Africa and Cameroun, respectively [30,31]. A prevalence of 26% has also been reported in Nigeria and Ghana, with *E. coli* being the most common bacteria isolate [30,32].

Jagadeesan et al. [28] and Bonadio et al. [33] have also presented similar information on diabetes and UTI among diabetic and non-diabetic individuals. However, the difference in prevalence of UTI, the causative bacteria, and their antimicrobial susceptibility patterns amongst diabetic patients and their non-diabetic caregivers at the same diabetes management center have not been established. Such information would help ascertain whether common UTI bacterial pathogens circulate among the DM patients and caregivers, and whether there exists any difference in their antimicrobial susceptibility patterns. This study therefore sought to present such information which will facilitate effective management among these group of individuals. 

## 2. Materials and Methods

### 2.1. Study Design, Site and Sampling

This was a prospective cross-sectional study design made up of two groups: diabetics patients and their caregivers who are non-diabetics. This design is similar to that of Mohammadshahi et al. [34]. The study was conducted between January and June 2020 and involved 100 diagnosed diabetic patients and a similar number of their caregivers who are non-diabetics, between the ages of 18 to 60 years. The diabetic participants had been diagnosed based on their glycated haemoglobin (HbA1c) and fasting plasma glucose (FPG) test results [1]. These patients had consistently displayed high plasma glucose levels, and had shown HbA1c assay results that classified their diabetes status as: HbA1c of 6.0–8.0% as good diabetes, 8.1–10.0% as fair diabetes, and >10.0 as poor diabetes [35,36,37]. The American Diabetes Association (ADA) and the European Association for the Study of Diabetes (EASD) recommend a HbA1c cut-off point of 7.0% (53.0 mmol/mol) as optimal [35,36,37]. 

In Ghana, however, most HbA1c assay results from reputable laboratories such as Synlab Ghana Limited, MDS Lancet Laboratories Limited and Greater Accra Regional Hospital laboratory, which work closely with health facilities and clinicians, come with a glycaemic control guideline (reference range) as described [37]. In this study, individuals with transient elevated plasma glucose levels were not enrolled, as well those on antibiotics. Sample collection was conducted among patients attending their normal diabetic reviews at the National Diabetes Management and Research Centre of the Korle-Bu Teaching Hospital. The non-diabetic caregivers were either workers or healthcare students on internship at the National Diabetes Management and Research Centre of the Hospital, who consented and were willing to be part of the study. Urine samples of about 5 mL were collected from the study participants by the “clean catch mid-stream method” for obtaining urine for culture. Samples were collected in sterile containers provided for use by the patients, and after they were adequately instructed on how to avoid contamination. The samples were then transported on ice pack under cold conditions to the Microbiology Laboratory of the University of Ghana Medical School, for microbiological analyses. The demographics such as age and gender of the enrolled diabetic cases were obtained from the folders/records of the patients while questionnaires were used to gather information from the non-diabetic individuals.

### 2.2. Sample Sizes Determination

Asamoah-Boaheng et al. [8] have described the prevalence of diabetes mellitus among adult Ghanaians to be 6.46%. Based on this, the sample size was calculated using Cochrane’s formula, *n* = Z^2^ × *p*(1 − *p*)/*m*^2^. Therefore, the minimum sample size was determined as described below: 

Description: *n* = minimum sample size; *z* = confidence level at 95% (standard value of 1.96); *m* = margin of error at 5% (standard value of 0.05); *p* = expected proportion of population based on prevalence from Asamoah-Boaheng et al. [8], = 6.46%. or 0.0646%.

*n* = 1.96^2^ × 0.0646 (1 − 0.0646)/(0.05)^2^

*n* = 0.2468 × 0.9354/0.0025 = 92 participants

Therefore, the minimum sample size required was 92 individuals. However, the convenience sampling technique was employed to collect samples consecutively from 100 consenting diabetic patients and a similar number of their caregivers who all fulfilled the inclusion criteria.

### 2.3. Identification and Antimicrobial Susceptibility Testing of Bacteria Isolates

The type of bacterial isolates was assessed by growth on cysteine lactose electrolyte deficient (CLED) media (Becton, Dickinson & Co, Franklin Lakes, NJ, USA). A loopful of the urine sample was picked and inoculated onto the CLED agar and incubated at 37 °C for 18–24 h. Mixed colonies were sub-cultured to obtain pure colonies. The identification of the grown colonies was based on colonial morphology, Gram staining, and a battery of biochemical reactions such as the triple sugar iron test, catalase test, urease test, indole test, and citrate utilization test [38,39,40,41,42,43]. Identified colonies were confirmed with the Bruker MALDI Biotyper^®^ IVD, according to the manufacturer’s instructions. 

The identified bacterial colonies were purified and, using the Kirby Bauer method, their susceptibility patterns were determined for different antibiotics that seem to be common on the Ghanaian market and among the commonly used in the treatment of UTIs in the country [12,44]. The antibiotics tested included ampicillin (AMP, 10 µg), nitrofurantoin (NIT, 200 µg), penicillin G (PG, 10 µg), cefuroxime (CXM, 30 µg), ceftriaxone (CRO, 30 µg), cefotaxime (CTX, 30 µg), ciprofloxacin (CIP, 5 µg), levofloxacin (LEV, 15 µg), ceftazidime (CEZ, 10 µg), amikacin (AMK, 30 µg), oxacillin (OX, 10 µg), gentamicin (GM, 10 µg) and trimethoprim-sulfamethoxazole (TS, 1.25/23.75 µg) (Oxoid Ltd., Basingstoke, UK). The antibiotic susceptibility testing procedure employed is briefly described as follows. The test organism was emulsified in peptone water until the turbidity was comparable with 0.5% McFarland’s standard. A loopful of the suspension was transferred onto a Mueller–Hinton agar plate (Becton, Dickinson & Co, Franklin Lakes, NJ, USA), and then a sterile cotton swab was used to streak the entire surface of the plate. Sterile forceps were used to apply the antibiotic discs to the surface of the agar plate, which was incubated at 37 °C for 18–24 h. Zone diameters around the antibiotic discs were measured and classified as sensitive or resistant based on the NCLS break point system [41]. *Escherichia coli NCTC 10418* was used as control strain in checking the efficacy/potency of discs for the susceptibility test.

### 2.4. Statistical Analysis

Data obtained were stored in Microsoft Excel and analyzed using the Statistical Products and Services Solutions (IBM^®^ SPSS^®^ version 25.0). Data were summarized by determining frequencies of isolates, as well as whether the difference in isolates between diabetic patients and non-diabetic individuals was significant. A *p*-value ˂ 0.05 was considered statistically significant.

### 2.5. Ethical Clearance

Ethical approval was sought from the Ethics and Protocol Review Committee of the School of Biomedical and Allied Health Sciences (SBAHS), University of Ghana, Accra, Ghana (Ethics Identification Number: SBAHS/AA/MLAB/10872318/20215-2016). Permission was also sought from the National Diabetes Management and Research Centre of the Korle-Bu Teaching Hospital. Informed consent was sought from all participants including the non-diabetic individuals. 

## 3. Results

### 3.1. Sociodemographic Data of Study Participants

In this study, 100 urine samples each were obtained from diabetics and non-diabetics patients, making a total of 200 patients. The diabetic patients were made up of 82 females and 18 males whiles the non-diabetics comprised 66 females and 44 males. The age of participants in both groups ranged from 18 to 60 years (Table 1). 

### 3.2. Prevalence of Urinary Tract Bacterial Pathogens in the Diabetic and Non-Diabetic Individuals

Among the diabetic and non-diabetic individuals, urinary tract bacterial pathogens were observed in 28.0% and 26.0% of samples, respectively (Table 2). The organisms were in the following proportions for diabetic and non-diabetic individuals, respectively: *E. coli* (14/28, 50% and 8/26, 30.7%), *S. aureus* (2/28, 7.1% and 4/26, 15.4%), *K. pneumoniae* (4/28, 14.3% and 8/26, 30.7%), *K. ozoenae* (2/28, 7.14% and 0/26, 0%), *K. oxytoca* (0/28, 0% and 4/26, 15.4%) and *C. urealyticum* (6/28, 21.4% and 2/26, 7.69%). The difference between the proportions of bacteria isolated was, however, not statistically significant (*p*-value = 0.894). Meanwhile, there were significant associations between sex (*p*-value = 0.011) and age (*p*-value = 0.034) with UTI among diabetic and non-diabetic individuals.

### 3.3. Susceptibility Profile of Bacteria Pathogens Isolated from Diabetic and Non-Diabetics

With regards to the antibiotic pattern for isolates from the diabetic patients, nitrofurantoin, cefuroxime, ceftriaxone, and cefotaxime recorded the highest potency, with all the isolates being 100% susceptible to these antibiotics (Table 3). Among the isolates, individually, *K. pneumonia* showed high susceptibility (100%) to all the antibiotics tested except ampicillin (50%). *S. aureus* was susceptible to all the antibiotics but showed high resistance (100%) to levofloxacin, trimethoprim, and amikacin (Table 3). *Corynebacterium urealyticum* (66.7–100%) and *E. coli* (57.1–100%), on the other hand, showed varying degrees of susceptibility to the antibiotics; however, *E. coli* displayed resistance to trimethoprim. *K. ozoenae* was susceptible to all the antibiotics except ceftazidime (Table 3). Multidrug resistance was displayed mainly by *S. aureus*, where it showed high resistance (100%) to three antibiotics (Table 3).

The antibiotic pattern for isolates from the non-diabetic individuals showed similar patterns to those of the diabetic patients (Table 4). However, ten out of the thirteen antibiotics tested recorded the highest potency, with all the isolates being 100% susceptible to these antibiotics (Table 4). Among the individual isolates, *K. pneumonia* showed high susceptibility (100%) to all the antibiotics tested except ampicillin with 100% resistance (Table 4). *S. aureus* was also susceptible to all the antibiotics but, unlike that of the diabetic patients, it was resistant (100%) to only trimethoprim. *C. urealyticum* (100%) and *E. coli* (75–100%) also showed high susceptibility to the antibiotics. *K. oxytoca*, which was isolated from the non-diabetic individuals instead of *K. ozoenae*, was also susceptible to all the antibiotics. No multidrug resistance was displayed in the isolates from the non-diabetic patients.

**Figure 1:**
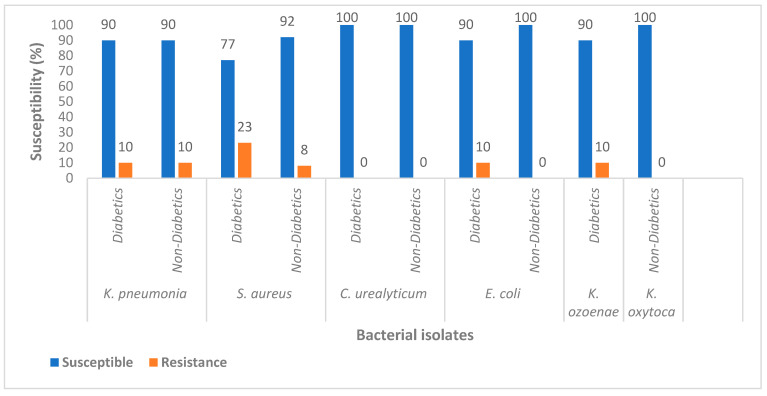
Overall susceptibility of bacterial isolates. *E. coli*: *Escherichia coli*, *S. aureus*: *Staphylococcus aureus*, *K. pneumoniae*: *Klebsiella pneumoniae*, *K. oxytoca*: *Klebsiella oxytoca*, *K. ozoenae*: *Klebsiella ozoenae*, *C. urealyticum*: *Corynebacterium urealyticum*.

In terms of the overall susceptibility of pathogens, *S. aureus* showed 77% susceptibility in the diabetic patients against 92% for the non-diabetic individuals. *E. coli* showed 90% susceptibility in diabetics as compared to 100% in non-diabetic individuals. Complete (100%) susceptibility was observed for *C. urealyticum* in both groups, while *K. pneumonia* showed 90% susceptibility in both groups. *K. ozoenae* and *K. oxytoca* showed 90% and 100% susceptibility in diabetics and non-diabetics, respectively (Figure 1).

Using *E. coli* as a dominant isolate, it was observed that there were no significant differences (*p*-values greater that 0.05) in susceptibility patterns among diabetics and non-diabetic individuals (Table 5).

## 4. Discussion

Ghana is a fast-growing lower middle-income country with a dense urban population [45]. Many patients do not undergo regular diagnosis for diabetes mellitus (DM) unless they show symptoms. Generally, UTIs have been observed in more females compared to males (including diabetics) [30] and this was similarly observed in the current study. The link between diabetes mellitus and urinary tract infections is increasingly being reported [27,28,33].

In this study, among the diabetic and non-diabetic individuals, urinary tract bacterial pathogens were observed in 28.0% and 26.0% of patients, respectively. This is similar to the study by Ekwealor et al. [46] where the overall prevalence of urinary tract infections among diabetic patients was 27.39%; however, contrary to the similarity observed in the current study, prevalence was significantly higher in diabetics than in non-diabetics (17.83%). This could be due to the difference in study populations. In the study by Ekwealor et al. [46], the non-diabetic individuals comprised both hospital outpatients and inpatients, but the non-diabetics in the current study consisted of caregivers (i.e., workers or healthcare students on internship at the National Diabetes Management and Research Centre of the Hospital). 

In another study with findings incomparable to that of the current study, Worku et al. [13] reported significant bacteriuria of 9.8% among diabetic patients. Other studies [27,47] have also reported lower prevalence rates of 19.5% and 13.8%, respectively, among diabetic patients. Meanwhile, some other researchers have reported higher prevalence of urinary tract infections among diabetic patients and non-diabetics with prevalence rates of 40% to 50.7% being observed among diabetics [48,49].

The high prevalence rate in some studies could be attributed to the emergence of antibiotic-resistant bacteria that cause urinary tract infections in diabetics in that population, as well as the study location and other risk factors that predispose diabetics to UTIs [46]. In a study by Ekwealor et al. [46], gender and previous history of UTI were found to have significant associations with urinary tract infection and this study also found gender and age to be associated with UTI among the groups.

Bacteriological studies usually reveal the involvement of Gram-negative enteric organisms that commonly cause urinary tract infections, such as *E. coli*, Klebsiella species, and the Proteus species [50].

Similarly, the predominant number of pathogens isolated in this study were Gram-negative pathogens rather than Gram-positive pathogens. *Escherichia coli*, *Klebsiella pneumoniae*, *Klebsiella ozoenae*, *Staphylococcus aureus* and *Cornyebacterium urealyticum* were identified amongst the diabetic patients with *Escherichia coli* being the most predominant bacteria, followed by *Cornyebacterium urealyticum*. A study conducted in Iran by Mohammadshahi et al. [51] also showed *E. coli* to be the most common uropathogen isolated from both diabetic and non-diabetic individuals. In that study, other common organisms in diabetic and non-diabetic individuals were *Staphylococcus aureus* and coagulase negative Staphylococcus. *E. coli* has been identified as the commonest UTI bacteria isolate among diabetics in Nigeria and Ghana [30,32]. The isolates *Staphylococcus* spp., *Escherichia coli* and *Klebsiella* spp. have been identified among common uropathogens among diabetic patients with urinary tract infection [52]. In this study, similar types of isolates were observed among the non-diabetic individuals, with the difference being *Klebsiella ozoenae* observed in diabetics and *Klebsiella oxytoca* in non-diabetics.

The similarities could be due to the reason that both the diabetic patients and non-diabetic individuals are all from the same center in terms of one being patients and the other being caregivers and students on clinical internships. In a review by Monteiro et al., [21] which looked to identify the potential health effects caused by bacterial contamination in the context of healthcare units, both in patients and in workers, concluded that bacteria transmission occurs mainly due to the contact between healthcare workers and patients or through the handling of or contact with contaminated instruments or surfaces, and the most common bacterial contaminants are *Escherichia coli*, *Pseudomonas aeruginosa*, *Staphylococcus* spp., *Staphylococcus aureus* and *Micrococcus luteus*. It was also established that the principal health effects of these contaminants are hospital-acquired infections and infections in immunocompromised people [21] and therefore it was not surprising that similar types of isolates were observed for diabetic and non-diabetic individuals at the same management center. Furthermore, Walsh and Collyns [20] described bacteria such as *Escherichia coli*, *Klebsiella* spp., *Pseudomonas* spp., *Enterobacter* spp., *Staphylococcus aureus*, and *Candida* spp. to be among the common range of aggressive pathogens involved in UTIs in diabetics.

*Cornyebacterium urealyticum* is a bacterial species of the *Cornyebacterium* genus that causes urinary tract infections amongst immunocompromised patients such as diabetics. *Klebsiella pneumonia* was the third highest, followed by *Klebsiella ozoenae* and *Staphylococcus aureus*. In a similar study from India, it was found that *E. coli* was the most commonly grown organism (64.3%), followed by *Staphylococcus aureus* (21.4%), and *Klebsiella pneumoniae* (14.3%) [53,54]. Diabetic patients with poor glycemic control have been found to have a higher tendency of *E. coli* adherence [55]. The prevalence of uropathogens was slightly higher in patients with DM with a percentage prevalence of 28% compared to the non-diabetics, with a percentage prevalence of 26%, and this difference was not significant. However, this outcome highlights the similarities in prevalence among the two groups studied. The prevalence of 28% for DM patients also compares to the prevalence of 26% which has been reported in Ghana and Nigeria [30,32].

Two species of Klebsiella were isolated from the study, *Klebsiella ozoenae* from diabetics and *Klebsiella oxytoca* from non-diabetics. *Klebsiella ozoenae* infections are more common in patients with diabetes even though they are rarely isolated. They are mostly treated with ciprofloxacin and intravenous aminoglycosides [56,57]. *Klebsiella oxytoca*, on the other hand, are usually referred to as ubiquitous and opportunistic in nature [58]. Outbreaks can occur in patients who are being treated with antibiotics as they tend to cause urinary tract infections in patients who are not even diabetics once the immune system becomes compromised [59]. *K. oxytoca* infection is treated with ceftazidime and the mortality rate at 14 days of its bacteremia has been reported to be 21% [60].

The antibiotic susceptibility pattern of the etiological agents in this study also revealed clear similarities among isolates from DM patients and non-diabetic individuals, with most of the bacteria isolates being highly susceptible to the antibiotics tested. Antibiotics such as nitrofurantoin, cefuroxime, ceftriaxone and cefotaxime recorded the highest potency for isolates from both groups of participants. The efficacy of these antibiotics might be due to the reason that these drugs are not easily available and/or are relatively expensive compared to others in the study area. Therefore, these drugs could be considered as therapeutic options in the empirical treatment of UTIs in the study subjects. However, a high level of resistance has been observed in some of these antibiotics, especially cefuroxime, as well as high multidrug resistance for Gram-negative bacteria [13], which was not the case in this study. Furthermore, unlike in this study, multidrug resistance was reported in 81.1% of the isolated bacteria in a study by Woldemariam et al. [61].

Nitrofurantoin is described as an antibiotic medication that is used for the treatment of uncomplicated lower urinary tract infections [62]. It is said to be effective against most Gram-positive and Gram-negative organisms. Clinical cure rates in nitrofurantoin have been reported to range from 51 to 94% depending on the length of follow-up, and bacteriological cure rates range from 61 to 92% [63]. Therefore, it is not surprising that it is among the most effective antibiotics in this study. 

A study in Germany on empiric antibiotic therapy in urinary tract infection in patients with risk factors for antibiotic resistance showed susceptibilities of 98% and 100% for cefuroxime and ceftazidime, respectively, for patients with no risk factors, while for patients with one risk factor, susceptibility rates of 78% for cefuroxime and 83% for ceftazidime were recorded [64]. 

Identifying that numerous antibiotics which are among the commonly prescribed antibiotics in Ghana are effective against UTI isolates from DM patients and non-diabetic individuals is a positive result and would help in effective managements of UTI in such patients to avoid diabetes–UTI complications [65].

Among isolates from DM patients, *S. aureus* showed high resistance (100%) to levofloxacin, trimethoprim and amikacin and *E. coli* displayed resistance to trimethoprim, while for non-diabetic individuals, resistance was observed for only trimethoprim against *S. aureus* and *K. pneumonia* was resistant to ampicillin. This is not surprising since *S. aureus* is among the common bacteria that have been observed to display multidrug resistance in the treatment of UTI [66]. This bacterium has been described among those that develop MDR due to biofilm formation which increases the inability of antibiotics to penetrate biofilms playing a key role in such behavior [67]. Similarly, resistance of *E. coli* to some antibiotics used in the treatment of UTI has been reported [68] and it has been said that urinary tract infection due to *E. coli* may be difficult to treat empirically due to high resistance to commonly used antibiotics. However, it is promising that the *E. coli* isolates in the current study were susceptible to most of the antibiotics tested. 

## 5. Conclusions

This study has shown that similarities exist in prevalence of UTI, the causative bacteria, and their antimicrobial susceptibility patterns amongst diabetic patients and non-diabetic individuals at the same diabetes management center. The bacteria isolates were mostly susceptible to the antibiotics tested and this is a good observation. Data from this study will help in the management of UTI among these individuals.

## 6. Limitations

Although some limitations can be identified in the study, these did not significantly affect the outcome and interpretations. For example, the DM cases were not classified into types, i.e., type 1 or 2. Further studies may consider that and ascertain significant associations with such classifications. Nonetheless, the outcomes from this study underscore the fact that there exist similarities in prevalence of UTI, the causative bacteria, and their antimicrobial susceptibility patterns amongst diabetic patients and non-diabetic individuals at the same diabetes management center.

## Figures and Tables

**Table 1:** Age and gender of study participants.

	Diabetics		Non-Diabetics	
Age Range	Sex		Sex	
	Female	Male	Female	Male
18–30	10	2	4	4
31–40	20	6	18	12
41–60	52	10	44	18

**Table 2:** Bacterial isolates identified from diabetic and non-diabetic patients.

Bacteria	Diabetic Group		Non-Diabetic Group		*p*-Value
	*n*	%	*n*	%	
*E. coli*	14	50	8	30.77	0.894
*S. aureus*	2	7.14	4	15.38	
*K. pneumoniae*	4	14.29	8	30.77	
*K. ozoenae*	2	7.14	0	0	
*K. oxytoca*	0	0	4	15.38	
*C. urealyticum*	6	21.43	2	7.69	
Total	28	100	26	100	

*E. coli*: *Escherichia coli*, *S. aureus*: *Staphylococcus aureus*, *K. pneumoniae*: *Klebsiella pneumoniae*, *K. oxytoca*: *Klebsiella oxytoca*, *K. ozoenae*: *Klebsiella ozoenae*, *C. urealyticum*: *Corynebacterium urealyticum*.

**Table 3:** In vitro antimicrobial susceptibility pattern of the bacterial isolates for diabetic patients.

Antibiotics	Pattern (S or R)	*K. pneumonia* (*n* = 4)	*K. ozoenae* (*n* = 2)	*S. aureus* (*n* = 2)	*C. urealyticum* (*n* = 6)	*E. coli* (*n* = 14)
NIT	S R	4 (100) 0 (0)	2 (100) 0 (0)	2 (100) 0 (0)	6 (100) 0 (0)	14 (100) 0 (0)
AMP	S R	2 (50) 2 (50)	2 (100) 0 (0)	2 (100) 0 (0)	6 (100) 0 (0)	8 (57.1) 6 (42.9)
TS	S R	4 (100) 0 (0)	2 (100) 0 (0)	0 (0) 2 (100)	4 (66.7) 2 (33.3)	6 (42.9) 8 (57.1)
GM	S R	4 (100) 0 (0)	2 (100) 0 (0)	2 (100) 0 (0)	6 (100) 0 (0)	12 (85.7) 2 (14.3)
CXM	S R	4 (100) 0 (0)	2 (100) 0 (0)	2 (100) 0 (0)	6 (100) 0 (0)	14 (100) 0 (0)
CRO	S R	4 (100) 0 (0)	2 (100) 0 (0)	2 (100) 0 (0)	6 (100) 0 (0)	14 (100) 0 (0)
CTX	S R	4 (100) 0 (0)	2 (100) 0 (0)	2 (100) 0 (0)	6 (100) 0 (0)	14 (100) 0 (0)
CIP	S R	4 (100) 0 (0)	2 (100) 0 (0)	2 (100) 0 (0)	6 (100) 0 (0)	10 (71.4) 4 (28.6)
LEV	S R	4 (100) 0 (0)	2 (100) 0 (0)	0 (0) 2 (100)	4 (66.7) 2 (33.3)	8 (57.1) 6 (42.9)
CEZ	S R	4 (100) 0 (0)	0 (0) 2 (100)	2 (100) 0 (0)	4 (66.7) 2 (33.3)	12 (85.7) 2 (14.3)
PG	S R	NT	NT	2 (100) 0 (0)	6 (100) 0 (0)	NT
AMK	S R	4 (100) 0 (0)	2 (100) 0 (0)	0 (0) 2 (100)	6 (100) 0 (0)	14 (100) 0 (0)
OX	S R	NT	NT	2 (100) 0 (0)	6 (100) 0 (0)	NT

NT means not tested, S means susceptible, R means resistant; *E. coli*: *Escherichia coli*, *S. aureus*: *Staphylococcus aureus*, *K. pneumoniae*: *Klebsiella pneumoniae*, *K. oxytoca*: *Klebsiella oxytoca*, *K. ozoenae*: *Klebsiella ozoenae*, *C. urealyticum*: *Corynebacterium urealyticum*, AMP: ampicillin, NIT: nitrofurantoin, PG: penicillin G, CXM: cefuroxime, CRO: ceftriaxone, CTX: cefotaxime, CIP: ciprofloxacin, LEV: levofloxacin, CEZ: ceftazidime, AMK: amikacin, OX: oxacillin, GM: gentamicin and TS: trimethoprim-sulfamethoxazole.

**Table 4:** In vitro antimicrobial susceptibility pattern of the bacteria isolates for non-diabetic individuals.

Antibiotics	Pattern (S or R)	*K. Pneumonia* (*n* = 8)	*K. Oxytoca* (*n* = 4)	*S. aureus* (*n* = 4)	*C. urealyticum* (*n* = 2)	*E. coli* (*n* = 8)
NIT	S R	8 (100) 0 (0)	4 (100) 0 (0)	4 (100) 0 (0)	2 (100) 0 (0)	8 (100) 0 (0)
AMP	S R	0 (0) 8 (100)	4 (100) 0 (0)	4 (100) 0 (0)	2 (100) 0 (0)	8 (100) 0 (0)
TS	S R	8 (100) 0 (0)	4 (100) 0 (0)	0 (0) 4 (100)	2 (100) 0 (0)	8 (100) 0 (0)
GM	S R	8 (100) 0 (0)	4 (100) 0 (0)	4 (100) 0 (0)	2 (100) 0 (0)	8 (100) 0 (0)
CXM	S R	8 (100) 0 (0)	4 (100) 0 (0)	4 (100) 0 (0)	2 (100) 0 (0)	8 (100) 0 (0)
CRO	S R	8 (100) 0 (0)	4 (100) 0 (0)	4 (100) 0 (0)	2 (100) 0 (0)	8 (100) 0 (0)
CTX	S R	8 (100) 0 (0)	4 (100) 0 (0)	4 (100) 0 (0)	2 (100) 0 (0)	8 (100) 0 (0)
CIP	S R	8 (100) 0 (0)	4 (100) 0 (0)	4 (100) 0 (0)	2 (100) 0 (0)	8 (100) 0 (0)
LEV	S R	8 (100) 0 (0)	4 (100) 0 (0)	4 (100) 0 (0)	2 (100) 0 (0)	8 (100) 0 (0)
CEZ	S R	8 (100) 0 (0)	4 (100) 0 (0)	4 (100) 0 (0)	2 (100) 0 (0)	6 (75) 2 (25)
PG	S R	NT	NT	4 (100) 0 (0)	2 (100) 0 (0)	NT
AMK	S R	8 (100) 0 (0)	4 (100) 0 (0)	4 (100) 0 (0)	2 (100) 0 (0)	8 (100) 0 (0)
OX	S R	NT	NT	4 (100) 0 (0)	2 (100) 0 (0)	NT

NT means not tested, S means susceptible, R means resistant; *E. coli*: *Escherichia coli*, *S. aureus*: *Staphylococcus aureus*, *K. pneumoniae*: *Klebsiella pneumoniae*, *K. oxytoca*: *Klebsiella oxytoca*, *K. ozoenae*: *Klebsiella ozoenae*, *C. urealyticum*: *Corynebacterium urealyticum*, AMP: ampicillin, NIT: nitrofurantoin, cotrimoxazole, CXM: cefuroxime, CRO: ceftriaxone, CTX: cefotaxime, CIP: ciprofloxacin, LEV: levofloxacin, CEZ: ceftazidime, AMK: amikacin, OX: oxacillin, GM: gentamicin and TS: trimethoprim-sulfamethoxazole.

**Table 5:** Susceptibility pattern of *E. coli* isolates from diabetic and non-diabetic individuals with UTI to different antibiotics.

Antibiotics	S/R	Diabetics, *n* (%) *n* = 14	Non-Diabetics, *n* (%) *n* = 8	*p*-Value	Total, *n* (%)
NIT	S R	14 (100) 0 (0)	8 (100) 0 (0)	0.702	22 (100) 0 (0)
AMP	S R	8 (57.1) 6 (42.9)	8 (100) 0 (0)	0.106	16 (100) 6 (0)
TS	S R	6 (42.9) 8 (57.1)	8 (100) 0 (0)	0.076	14 (100) 8 (0)
GM	S R	12 (85.7) 2 (14.3)	8 (100) 0 (0)	0.825	20 (100) 2 (0)
CXM	S R	14 (100) 0 (0)	8 (100) 0 (0)	0.702	22 (100) 0 (0)
CRO	S R	14 (100) 0 (0)	8 (100) 0 (0)	0.702	22 (100) 0 (0)
CTX	S R	14 (100) 0 (0)	8 (100) 0 (0)	0.702	22 (100) 0 (0)
CIP	S R	10 (71.4) 4 (28.6)	8 (100) 0 (0)	0.321	18 (100) 4 (0)
LEV	S R	8 (57.1) 6 (42.9)	8 (100) 0 (0)	0.106	16 (100) 6 (0)
CEZ	S R	12 (85.7) 2 (14.3)	6 (75) 2 (25)	0.530	18 (75) 4 (25)
PG	S R	NT	NT		NT
AMK	S R	14 (100) 0 (0)	8 (100) 0 (0)	0.702	22 (100) 0 (0)
OX	S R	NT	NT		NT

NT means not tested, S means susceptible, R means resistant, AMP: ampicillin, NIT: nitrofurantoin, cotrimoxazole, CXM: cefuroxime, CRO: ceftriaxone, CTX: cefotaxime, CIP: ciprofloxacin, LEV: levofloxacin, CEZ: ceftazidime, AMK: amikacin, OX: oxacillin, GM: gentamicin and TS: trimethoprim-sulfamethoxazole.

## Data Availability

All data supporting the results have been included in the paper.
